# To shift or to rotate? Comparison of acquisition strategies for multi-slice super-resolution magnetic resonance imaging

**DOI:** 10.3389/fnins.2022.1044510

**Published:** 2022-11-11

**Authors:** Michele Nicastro, Ben Jeurissen, Quinten Beirinckx, Céline Smekens, Dirk H. J. Poot, Jan Sijbers, Arnold J. den Dekker

**Affiliations:** ^1^imec-Vision Lab, Department of Physics, University of Antwerp, Antwerp, Belgium; ^2^μNEURO Research Centre of Excellence, University of Antwerp, Antwerp, Belgium; ^3^Lab for Equilibrium Investigations and Aerospace, Department of Physics, University of Antwerp, Antwerp, Belgium; ^4^Siemens Healthcare NV/SA, Groot-Bijgaarden, Belgium; ^5^Biomedical Imaging Group Rotterdam, Department of Radiology and Nuclear Medicine, Erasmus MC, Rotterdam, Netherlands

**Keywords:** magnetic resonance imaging, super-resolution, optimal experimental design, Bayesian estimation, image reconstruction

## Abstract

Multi-slice (MS) super-resolution reconstruction (SRR) methods have been proposed to improve the trade-off between resolution, signal-to-noise ratio and scan time in magnetic resonance imaging. MS-SRR consists in the estimation of an isotropic high-resolution image from a series of anisotropic MS images with a low through-plane resolution, where the anisotropic low-resolution images can be acquired according to different acquisition schemes. However, it is yet unclear how these schemes compare in terms of statistical performance criteria, especially for regularized MS-SRR. In this work, the estimation performance of two commonly adopted MS-SRR acquisition schemes based on shifted and rotated MS images respectively are evaluated in a Bayesian framework. The maximum a posteriori estimator, which introduces regularization by incorporating prior knowledge in a statistically well-defined way, is put forward as the estimator of choice and its accuracy, precision, and Bayesian mean squared error (BMSE) are used as performance criteria. Analytic calculations as well as Monte Carlo simulation experiments show that the rotated scheme outperforms the shifted scheme in terms of precision, accuracy, and BMSE. Furthermore, the superior performance of the rotated scheme is confirmed in real data experiments and in retrospective simulation experiments with and without inter-image motion. Results show that the rotated scheme allows regularized MS-SRR with a higher accuracy and precision than the shifted scheme, besides being more resilient to motion.

## 1. Introduction

Multi-slice (MS) super-resolution reconstruction (SRR) methods have been proposed to improve the inherent trade-off between spatial resolution, signal-to-noise ratio (SNR), and scan time in magnetic resonance imaging (MRI) (Greenspan, 2008; Plenge et al., 2012; Poot et al., 2012; Van Dyck et al., 2020; Askin Incebacak et al., 2021; Vis et al., 2021) and to replace direct 3D acquisition methods when these are not effective or possible, as is often the case, for example, in T2-weighted imaging (Greenspan, 2008; Plenge et al., 2012). MS-SRR consists in estimating a 3D isotropic high-resolution (HR) image, hereafter referred to as MS-SRR image, from a series of anisotropic MS images with a low through-plane resolution, where each of the low resolution MS images is acquired in a distinct fashion to ensure that the acquired dataset contains complementary resolution information about the HR image to be reconstructed (Van Reeth et al., 2012).

Two MS-SRR acquisition strategies are commonly applied that have been demonstrated to fulfill this requirement. According to these strategies, the MS images are acquired with (*i*) sub-voxel shifts in the through-plane direction (Greenspan et al., 2002), (*ii*) slice orientations rotated around a common frequency (Shilling et al., 2008, 2009) or phase-encoding axis (Poot et al., 2012; Van Steenkiste et al., 2015, 2016). These acquisition strategies were first compared in the same works in which the MS-SRR rotated acquisition scheme was proposed (Shilling et al., 2008, 2009). In Shilling et al. (2009), the shifted and rotated MS-SRR acquisition schemes were compared in terms of the mean squared error (MSE) and sharpness of the MS-SRR image using simulation and real data experiments, respectively. In these experiments, iterative algorithms based on the projection onto convex sets (POCS) method (Stark and Oskoui, 1989) were adopted that converge to a Maximum Likelihood solution of the non-regularized MS-SRR problem. Results with the rotated scheme showed a sharper MS-SRR image with a lower MSE than the shifted scheme. However, since MS-SRR estimation involves solving an ill-conditioned inverse problem, unregularized estimators, although unbiased, often have an unacceptably high variance. Furthermore, when unregularized estimators are used, small modeling errors may lead to reconstruction artifacts that prevent the MS-SRR image's diagnostic use (Khattab et al., 2020). These artifacts can be minimized by regularizing the MS-SRR problem, as qualitatively shown by Shilling et al. (2008). Nevertheless, up to now, to the authors' knowledge, no statistical evaluation of MS-SRR acquisition strategies has been performed that takes account of the effect of regularization.

In this paper, a Bayesian framework is proposed that allows such an evaluation. Analytical expressions are derived for the accuracy, precision, and Bayesian mean squared error (BMSE) of the maximum a posteriori (MAP) estimator of the MS-SRR image, where the MAP estimator is put forward since it imposes regularization by incorporating prior knowledge of the MS-SRR image in a statistically well-defined way (Kay, 1993). The derived measures are then used as performance criteria to evaluate and compare the two MS-SRR acquisition strategies described above. The results of this evaluation are validated in Monte Carlo simulations and real data experiments. Furthermore, the impact of patient motion on the estimation performance of the acquisition protocols is investigated with retrospective simulations. The proposed analysis in a Bayesian framework, which is novel in super-resolution reconstruction, aims to pave the way toward optimal experiment design that allows to determine the optimal MS-SRR image acquisition strategy on a rigorous statistical basis. A preliminary version of this study, based on analytical calculations and numerical simulations only, was published as a proceedings paper (Nicastro et al., 2021).

## 2. Materials and methods

### 2.1. Theory

#### 2.1.1. MS-SRR forward model

Let $r\in {\mathbb{R}}^{N_{r}\times 1}$ represent the noiseless HR magnitude image to be reconstructed, with *N*_*r*_ the number of voxels. Furthermore, let $s_{n}\in {\mathbb{R}}^{N_{s}\times 1}$, with *n* = 1, …, *N*, be the *n*-th noiseless MS magnitude image, consisting of *N*_*s*_ voxels with an anisotropic voxel-size characterized by the anisotropy factor (*AF*), which is defined as the ratio of the through-plane resolution to the in-plane resolution. Next, let **θ**_*n*_ = (*t*_x_*n*__, *t*_y_*n*__, *t*_z_*n*__, ϕ_x_*n*__, ϕ_y_*n*__, ϕ_z_*n*__) be a rigid transformation parameter vector, where *t*_x_*n*__, *t*_y_*n*__, *t*_z_*n*__ are the translation parameters, expressed in mm, and ϕ_x_*n*__, ϕ_y_*n*__, ϕ_z_*n*__ the rotation angles around axis x, y, and z, respectively, expressed in degrees (°), with the center of rotation matching the center of the image. Then, *s*_*n*_ can be modeled as:


(1) $$\begin{array}{l} s_{n}\left(r\right)=\mathrm{DBM}\left(\theta_{n}\right)r, \end{array}$$

with $M\in {\mathbb{R}}^{N_{r}\times N_{r}}$, $B\in {\mathbb{R}}^{N_{r}\times N_{r}}$, and $D\in {\mathbb{R}}^{N_{s}\times N_{r}}$ linear operators that describe a geometric transformation, space-invariant blurring, and down-sampling, respectively. The motion operator ***M*** applies the rigid transformation defined by **θ**_*n*_. The blurring operator ***B*** models the point-spread function (PSF) of the MRI acquisition method. For MS acquisition methods, the PSF can be assumed separable and therefore modeled as the product of three 1D PSFs that are applied in the three orthogonal directions aligned with the MR image coordinate axes. The 1D PSFs in the in-plane directions (frequency- and phase-encoding) are modeled by a periodic sinc while the 1D PSF in the through-plane direction (slice-encoding) can be well-approximated by a Gaussian function having a full-width half maximum equal to the slice thickness (Van Reeth et al., 2012). The down-sampling operator ***D*** decimates the image in the slice-encoding direction by the factor *AF* by keeping only every *AF*-th voxel.

The MS images can be represented as a matrix-vector multiplication ***s***(***r***) = ***Ar***, where $s={\left[s_{1}^{T},\ldots ,s_{N}^{T}\right]}^{T}\in {\mathbb{R}}^{NN_{s}\times 1}$ and $A={\left[A_{1}^{T},\ldots ,A_{N}^{T}\right]}^{T}$ $\in {\mathbb{R}}^{NN_{s}\times N_{r}}$, with $A_{n}=DBM\left(\theta_{n}\right)\in {\mathbb{R}}^{N_{s}\times N_{r}}$. For an efficient implementation of the operators, we adopted the set of shears transformations method proposed by Poot et al. (2010).

#### 2.1.2. MAP estimator

Let $\tilde{s}\in {\mathbb{R}}^{NN_{s}\times 1}$ be the vector containing the voxel intensities of the acquired MS images. Since these MS images are disturbed by noise, $\tilde{s}$ is modeled as a random variable. MS-SRR involves the estimation of the HR image ***r*** from the MS images $\tilde{s}$. In this work, we follow a Bayesian estimation approach, in which the HR image ***r*** to be estimated is viewed as a realization of a random variable with distribution *p*(***r***). This so-called prior distribution *p*(***r***), being the joint distribution of the elements ${\left\{r_{i}\right\}}_{1}^{N_{r}}$ of ***r***, summarizes our initial state of knowledge about ***r*** before any images are acquired. After the images $\tilde{s}$ are acquired, our knowledge about ***r*** has increased and is now summarized by $p\left(r|\tilde{s}\right)$, which is known as the posterior probability density function (PDF) of ***r*** given $\tilde{s}$. According to Bayes' theorem, $p\left(r|\tilde{s}\right)$ can be expressed as


(2) $$p(r|\tilde{s})=\frac{p(\tilde{s}|r)p(r)}{p(\tilde{s})},$$

where $p\left(\tilde{s}|r\right)$ is the conditional PDF of $\tilde{s}$ given ***r*** and $p\left(\tilde{s}\right)$ is a normalization constant. When $p\left(\tilde{s}|r\right)$ is viewed as a function of ***r*** for a fixed data set $\tilde{s}$, it is called the likelihood function. The conditional PDF $p\left(\tilde{s}|r\right)$ of the magnitude images $\tilde{s}$ can be derived from the MS-SRR forward model described in Equation (1) and the assumed noise statistics.

Nowadays, parallel imaging is commonly adopted in MRI clinical practice to reduce scan time, allowing to reconstruct the magnitude MR image from sub-sampled acquisitions of the k-space from multiple coils. Generalized autocalibrating partially parallel acquisitions (GRAPPA) (Griswold et al., 2002) and sensitivity encoding (SENSE) (Pruessmann et al., 1999) are the two most often used parallel imaging approaches. When GRAPPA is adopted in combination with the spatially matched filter (SMF) technique (Walsh et al., 2000), or when SENSE is adopted, the reconstructed magnitude images $\tilde{s}$ follow a Rician distribution (Walsh et al., 2000; Aja-Fernández et al., 2014). Additionally, when the SNR is larger than 3, the Rician distribution can be well approximated by a Gaussian distribution (Gudbjartsson and Patz, 1995). Under these assumptions and assuming all voxel intensities to be statistically independent and the standard deviation of the noise σ to be temporally and spatially invariant, $p\left(\tilde{s}|r\right)$ can be expressed as follows:


(3) $$p(\tilde{s}|r)\propto \exp\left(-\frac{1}{2\sigma^{2}}{\left\|\tilde{s}-s(r)\right\|}_{2}^{2}\right).$$

Furthermore, the prior distribution *p*(***r***) can be modeled as a stationary Gaussian Markov random field (MRF) (Bardsley, 2013). This corresponds to the assumption of a multivariate Gaussian prior of the form:


(4) $$\begin{array}{l} p\left(r\right)\propto \exp\left(-\frac{1}{2}{\left(r-\bar{r}\right)}^{T}K^{-1}\left(r-\bar{r}\right)\right), \end{array}$$

which is parameterized by its mean $\bar{r}\in {\mathbb{R}}^{N_{r}\times 1}$ and precision (inverse-covariance) matrix $K^{-1}\in {\mathbb{R}}^{N_{r}\times N_{r}}$. This precision matrix, which is sparse and positive definite, encodes statistical correlations between the intensity of an HR image voxel and that of its neighboring voxels. Let $r_{\partial_{i}}\in {\mathbb{R}}^{N_{n}\times 1}$ be the voxel intensities from the neighborhood surrounding the *i*-th HR voxel, where *N*_*n*_ is the number of neighborhood voxels, and **∂**_*i*_ represents the neighborhood voxels' indices. Following the conditional auto-regression approach proposed by Besag (1974), we first define for each voxel *r*_*i*_ its neighborhood conditional PDF *p*(*r*_*i*_|***r***_**∂**_*i*__) as the conditional PDF of the intensity *r*_*i*_ of the *i*-th HR voxel given the intensities of its neighboring voxels ***r***_**∂**_*i*__. Then, we assume all neighborhood conditional PDFs to be Gaussian and of the form:


(5) $$\begin{array}{l} p\left(r_{i}|r_{\partial_{i}}\right)\propto \exp\left(-\frac{\lambda^{2}}{2}{\left(r_{i}-\underset{j\in \partial_{i}}{\sum }\alpha_{j}r_{j}\right)}^{2}\right), \end{array}$$

where $\alpha ={\left\{\alpha_{j}\right\}}_{1}^{N_{n}}\in {\mathbb{R}}^{N_{n}\times 1}$ is the vector of the so-called field potentials, or regression coefficients. Indeed, it follows from Equation (5) that the *i*-th HR image voxel intensity can be described by an auto-regressive model:


(6) $$\begin{array}{l} r_{i}=\underset{j\in \partial_{i}}{\sum }\alpha_{j}r_{j}+u_{i}, \end{array}$$

where *u*_*i*_ is a sample from a white Gaussian noise process with variance λ^2^. Furthermore, it can be demonstrated (Rue and Held, 2005) that Equation (5) holds if and only if the prior *p*(***r***) takes the form of Equation (4) with:


(7) $$K_{i,j}^{-1}=\lambda^{2}\{\begin{array}{cc} 1, & i=j,\\ -\alpha_{j} & j\in \partial_{i}. \end{array}$$

The maximum a posteriori (MAP) estimator $\hat{r}$ can then be obtained by maximizing $p\left(r|\tilde{s}\right)$ with respect to ***r***. Hence, by combining Equations (2) and (4), we obtain:


(8) $$\begin{array}{l} \hat{r}=\text{arg }\underset{r}{\text{max}}\text{ ln }p(r|\tilde{s})\\ \;=\text{arg }\underset{r}{\text{min}}\left(\frac{1}{2\sigma^{2}}\|\tilde{s}-Ar{\|}_{2}^{2}+\frac{1}{2}{\left(r-\bar{r}\right)}^{T}K^{-1}(r-\bar{r})\right). \end{array}$$

Note that the MAP estimator (Equation 8) corresponds with a regularized least squares estimator, which has the following closed-form solution:


(9) $$\begin{array}{l} \hat{r}={\left(\sigma^{-2}A^{T}A+K^{-1}\right)}^{-1}\left(\sigma^{-2}A^{T}\tilde{s}+K^{-1}\bar{r}\right). \end{array}$$

#### 2.1.3. Bayesian MSE

The BMSE of the MAP estimator described by Equation (9) is proposed as a performance criterion to compare the shifted and rotated MS-SRR acquisition schemes. Before deriving the BMSE, let us first define the component-wise MSE of $\hat{r}$ for a fixed value of ***r*** as:


(10) $$\begin{array}{l} \text{MSE}{\left(r\right)}_{j}={𝔼}_{\tilde{s}|r}{\left[\left(\hat{r}-r\right){\left(\hat{r}-r\right)}^{T}\right]}_{j,j}, \end{array}$$

with ${𝔼}_{\tilde{s}|r}\left[\cdot \right]$ the expectation operator over the conditional PDF $p\left(\tilde{s}|r\right)$.

The MSE described by Equation (10) can be decomposed as the sum of a variance term and a squared bias term (van den Bos, 2007):


(11) $$\begin{array}{l} \text{MSE}{\left(r\right)}_{j}=\Sigma_{j,j}+{\left[\beta \left(r\right)\beta^{T}\left(r\right)\right]}_{j,j}, \end{array}$$

where $\Sigma \in {\mathbb{R}}^{N_{r}\times N_{r}}$ and $\beta \in {\mathbb{R}}^{N_{r}\times 1}$ are the covariance matrix and the bias vector of $\hat{r}$, which are element-wise defined as:


(12) $$\begin{array}{l} \Sigma_{i,j}={𝔼}_{\tilde{s}}{\left[\left(\hat{r}-{𝔼}_{\tilde{s}}\left[\hat{r}\right]\right){\left(\hat{r}-{𝔼}_{\tilde{s}}\left[\hat{r}\right]\right)}^{T}\right]}_{i,j} \end{array}$$

and


(13) $$\begin{array}{l} \beta {\left(r\right)}_{j}={𝔼}_{\tilde{s}}{\left[\hat{r}\right]}_{j}-r_{j}, \end{array}$$

respectively. It can be shown straightforwardly that for the MAP estimator defined in Equation (9), the variance component and the bias component of the MSE assume the following closed-form expressions:


(14) $$\begin{array}{l} \Sigma =\sigma^{-2}QA^{T}AQ, \end{array}$$

(15) $$\beta \left(r\right)=QK^{-1}\left(r-\bar{r}\right),$$

with


(16) $$\begin{array}{l} Q={\left(\sigma^{-2}A^{T}A+K^{-1}\right)}^{-1}. \end{array}$$

Next, the component-wise BMSE of $\hat{r}$ is obtained as (Kay, 1993; Ben-Haim and Eldar, 2009):


$$\begin{array}{ll} \text{BMS}{\text{E}}_{j}\equiv & {𝔼}_{r,\tilde{s}}{\left[\left(\hat{r}-r\right){\left(\hat{r}-r\right)}^{T}\right]}_{j,j} \end{array}$$

(17) $$\begin{array}{ll} \;= & {𝔼}_{r}{\left[{𝔼}_{\tilde{s}|r}\left[\left(\hat{r}-r\right){\left(\hat{r}-r\right)}^{T}\right]\right]}_{j,j}=Q_{j,j}, \end{array}$$

with ${𝔼}_{r,\tilde{s}}\left[\cdot \right]$, 𝔼_***r***_[·], and ${𝔼}_{\tilde{s}|r}\left[\cdot \right]$ the expectation operators over the joint PDF $p\left(r,\tilde{s}\right)=p\left(\tilde{s}|r\right)p\left(r\right)$, the prior distribution *p*(***r***), and the conditional PDF $p\left(\tilde{s}|r\right)$, respectively. Note that it follows from Equations (10), (11), and (17) that, similar to the MSE, the BMSE can be decomposed into two terms:


$$\begin{array}{l} \text{BMS}{\text{E}}_{j}={𝔼}_{r}{\left[\Sigma \right]}_{j,j}+{𝔼}_{r}{\left[\beta \left(r\right)\beta^{T}\left(r\right)\right]}_{j,j} \end{array}$$

(18) $$\begin{array}{ll} \;= & \Sigma_{j,j}+{𝔼}_{r}{\left[\beta \left(r\right)\beta^{T}\left(r\right)\right]}_{j,j}, \end{array}$$

where the first term corresponds with the variance (Equation 12) of the MAP estimator (Equation 9), which does not depend on ***r***, and the second term corresponds with the expected value of the squared bias of the MAP estimator, where the expected value is taken over the prior distribution *p*(***r***). It can be shown that this second term assumes the following closed-form expression:


(19) $$\begin{array}{l} {𝔼}_{r}\left[\beta \left(r\right)\beta^{T}\left(r\right)\right]=QK^{-1}Q. \end{array}$$

### 2.2. MS-SRR protocols comparison

The comparison was conducted taking into account different combinations of *AF* and numbers of acquired MS images *N* for the MS-SRR protocols, but keeping the same scan time for all the experiments. Assuming an interleaved fast spin-echo (FSE) protocol, the acquisition time *t*_*acq*_ is given by (Bernstein et al., 2004):


$$\begin{array}{l} t_{acq}\approx N\cdot \text{TR}\cdot \left\lceil \frac{\text{FO}{\text{V}}_{se}}{\text{P}{\text{I}}_{acc}\cdot \text{ETL}\cdot \text{re}{\text{s}}_{se}}\right\rceil \end{array}$$

(20) $$\begin{array}{l} \;=N\cdot \text{TR}\cdot \left\lceil \frac{\text{FO}{\text{V}}_{se}}{\text{P}{\text{I}}_{acc}\cdot \text{ETL}\cdot \text{re}{\text{s}}_{ip}\cdot AF}\right\rceil , \end{array}$$

where TR is the repetition time, ⌈·⌉ is the round-up operator, FOV_se_ is the field of view in the slice-encoding direction, PI_*acc*_ is the parallel imaging acceleration factor, ETL the echo-train length, and res_*se*_ the slice thickness, where *AF* = res_*se*_/res_*ip*_, with res_*ip*_ being the in-plane voxel size. TR, FOV_*se*_, PI_*acc*_, ETL, and res_ip_ were kept fixed for all protocols. Furthermore, the ratio *N*/*AF* was set equal to 2. This choice ensures that all acquisition protocols require the same scan time, while at the same time the MS-SRR estimation problem is not under-determined (*N*/*AF* ≥ 1) (Shilling et al., 2009), and the k-space is efficiently sampled when the MS images are acquired with the rotated scheme $\left(N>\frac{\pi }{2}AF\right)$ (Van Steenkiste et al., 2015).

**Figure 1:**
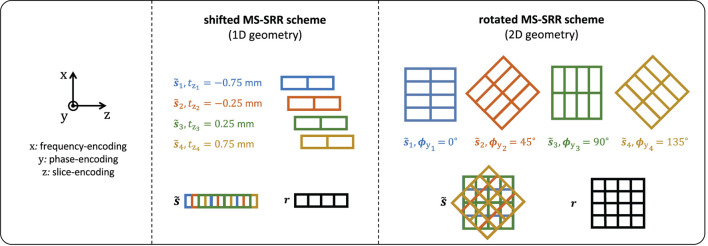
Acquisition geometries of the MS-SRR shifted and rotated schemes: *AF* = 2, *N* = 4, and res_*ip*_ = 1 mm.

The acquisition protocols included in the comparison are summarized in Table 1. An HR protocol representing a conventional HR MS acquisition with *AF* = 1 was included as a reference and repeated twice to equal the scan time of the SR protocols. In the SRrot protocols, the acquired images were rotated around the phase-encoding axis so that the phase-encoding axis is the same for all the MS images. The rigid transformation parameter vector for the *n*-th acquired MS image assumes the form **θ**_*n*_ = [0, 0, 0, 0, ϕ_y_*n*__, 0] and **θ**_*n*_ = [0, 0, *t*_z_*n*__, 0, 0, 0] for the SRrot and SRsh protocols, respectively. For the SRrot protocols, the rotation angles around the phase-encoding axis $\phi_{\text{y}}={\left\{\phi_{{\text{y}}_{n}}\right\}}_{n=1}^{N}$ were equidistantly chosen in the open interval [0, 180), with steps of 180/*N*, whereas, for the SRsh protocols, the shifts along the slice-encoding direction $t_{\text{z}}={\left\{t_{{\text{z}}_{n}}\right\}}_{n=1}^{N}$ were equidistantly chosen in the closed interval [−*AF*(*N* − 1)/(2*N*), *AF*(*N* − 1)/(2*N*)], with steps of *AF*/*N*. The acquisition geometries of the MS-SRR shifted and rotated schemes for the case of *AF* = 2, *N* = 4, and res_*ip*_ = 1 mm are represented in Figure 1.

**Table 1:** MS-SRR acquisition protocols included in the comparison.

**Protocols**	***AF***	***N***	***t*_z_, ϕ_y_**	** $\sigma_{sim}\left[10^{-3}\right]$ **	** $\hat{\sigma }\left[10^{-3}\right]$ **	${\hat{\text{SNR}}}^{*}$	***t*_*acq*_/*N***	***t*_*acq*_**
**HR** ^**^	1	2	–	27.02	27.02	7.16	4m 24s	8m 48s
SRsh_2_	2	4	*t*_z_ = [−0.75, −0.25, 0.25, 0.75] mm	13.51	–	–	–	–
SRsh_3_	3	6	*t*_z_ = [−1.25, −0.75, −0.25,					
			0.25, 0.75, 1.25] mm	9.01	–	–	–	–
**SRsh**_4_	4	8	*t*_z_ = [−1.75, −1.25, −0.75, −0.25,					
			0.25, 0.75, 1.25, 1.75] mm	6.75	7.23	26.74	1m 39s	13m 12s
SRrot_2_	2	4	$\phi_{\text{y}}={\left[0,45,90,135\right]}^{•}$	13.51	–	–	–	–
SRrot_3_	3	6	$\phi_{\text{y}}={\left[0,30,60,90,120,150\right]}^{•}$	9.01	–	–	–	–
**SRrot**_4_	4	8	ϕ_y_ = [0, 22.5, 45, 67.5,					
			90, 112.5, 135, 157.5]°	6.75	7.96	24.29	1m 39s	13m 12s

Each MS-SRR acquisition protocol is defined by the transformation parameter vector $t_{\text{z}}={\left\{t_{{\text{z}}_{n}}\right\}}_{n=1}^{\text{N}}$ or $\phi_{\text{y}}={\left\{\phi_{{\text{y}}_{n}}\right\}}_{n=1}^{\text{N}}$, where *t*_z_*n*__ describes a translation in the slice-encoding axis and ϕ_y_*n*__ describes a rotation around the phase-encoding axis, respectively, applied to the *n*-th MS image. Furthermore, the standard deviation of the noise used in both the computation of the BMSE-based metrics and in the Monte Carlo simulation experiments σ_*sim*_, the standard deviation of the noise $\hat{\sigma }$ and the $\hat{\text{SNR}}$ value estimated from the acquired MS images and used in the retrospective simulation experiments, the acquisition time per MS image *t*_*acq*_/*N* and the total acquisition time *t*_*acq*_ are reported. The protocols adopted in the real data experiments are indicated in bold.

$*\hat{\text{SNR}}$ values were calculated using the white matter tissue as a reference.

** The MS images were acquired with a limited FOV in the slice-encoding direction due to hardware constraints.

#### 2.2.1. MS-SRR protocols comparison in terms of BMSE

A simplified 2D framework is proposed to compare the MS-SRR protocols in terms of the BMSE and its separate squared bias and variance components by exploiting the inherent 1D acquisition geometry of the shifted scheme and the 2D acquisition geometry of the rotated scheme (Figure 1). The proposed framework allows to evaluate the MS-SRR problem in 2D by restricting the analysis to a single slice along the phase encoding direction and neglecting the blurring component in the same direction. This simplification is expected to reduce the computational complexity and memory consumption without influencing the results of our comparison, since this blurring affects all protocols equivalently.

##### 2.2.1.1. 2D training and validation datasets

The prior hyperparameters **α**, $\bar{r}$, and λ, introduced in Section 2.1.2, were learned from a training dataset composed of 100 noiseless HR 2D T2-weighted magnitude brain images of resolution 1 × 1 mm^2^ and size 217 × 217. These images were simulated from 10 of the 20 3D anatomical brain models available in the Brainweb database (Cocosco et al., 1997) as follows. Each anatomical model consists of a set of discrete 3D tissue membership volumes, one for each tissue class: background, cerebrospinal fluid, gray matter, white matter, fat, muscle, skin, skull, blood vessels, connective tissue, dura mater, and bone marrow. T2-weighted brain volumes were generated from the 3D anatomical models by applying the spin-echo decay model (Jung and Weigel, 2013):


(21) $$\begin{array}{l} r_{i}=\rho_{i}\left(1-e^{-\frac{\text{TR}}{\text{T}1_{i}}}\right)e^{-\frac{\text{TE}}{\text{T}2_{i}}}, \end{array}$$

where *r*_*i*_ is the *i*-th voxel intensity, TE is the echo-time, and ρ_*i*_, T1_*i*_, and T2_*i*_ are the *i*-th voxel proton density, longitudinal relaxation, and transverse relaxation values, fixed for each tissue to the mean value of its respective distribution at 3T reported by Sabidussi et al. (2021). TR and TE were independently sampled for each image within the training dataset from uniform distributions with ranges [10, 14] s and [80, 120] ms, respectively. A series of 2D slices of resolution 1 × 1 mm^2^ was then simulated from three orthogonal acquisition planes (sagittal, transverse, and coronal), by down-sampling and then slicing the T2-weighted volumes. The thus obtained images, representing independent realizations of ***r***, and characterized each by a unique morphology, orientation and contrast, compose the 2D training dataset. The same approach was used to simulate the *N*_*v*_ = 100 additional 2D T2-weighted magnitude brain images that compose the 2D validation dataset from the 10 remaining anatomical brain models.

##### 2.2.1.2. 2D MRF prior hyperparameters

The prior hyperparameters **α**, $\bar{r}$, and λ were estimated from the image voxels within the 2D training dataset and their respective 2D neighborhoods of size *p* × *p*, with $p=\sqrt{N_{n}}$. The parameters **α** and λ were estimated by solving the Yule-Walker equations using a least-squares estimator (Eshel, 2011). The kernel size of the voxel neighborhoods was defined by testing increasing odd values of *p*, starting from *p* = 3, until a stable estimate of λ was found. We defined the estimate $\hat{\lambda }$ of λ to be stable if the relative difference of $\hat{\lambda }$ for the current value of *p* with respect to the value of $\hat{\lambda }$ for the previous value of *p* was <1%. The condition was met for *p* = 11. All the elements of the prior mean $\bar{r}$ were set equal to the mean intensity of the images within the training dataset, thus ensuring the prior is translation-invariant.

##### 2.2.1.3. Quantitative metrics

The BMSE and its separate variance and squared bias components were computed using the closed-form expressions introduced in Section 2.1.3. For each acquisition protocol, since the SNR is expected to increase proportionally with the slice thickness, the standard deviation of the noise σ_*sim*_ was set to match $\hat{\sigma }/AF$, where $\hat{\sigma }$ is the standard deviation of the noise estimated from the (pre-processed) MS images acquired in the real data experiments using the HR protocol. The real data experiments and the estimation of the standard deviation of noise $\hat{\sigma }$ from the acquired MS images will be further described in Section 2.2.3.1. The values of σ_*sim*_ and $\hat{\sigma }$ are reported in Table 1.

Voxel-wise BMSE-based metrics were calculated as follows:


(22) $$\begin{array}{l} \text{BRMS}{\text{E}}_{j}=\sqrt{Q_{j,j}}, \end{array}$$

(23) $$\begin{array}{l} \text{S}{\text{D}}_{j}=\sqrt{\sigma^{-2}{\left[QA^{T}AQ\right]}_{j,j}}, \end{array}$$

(24) $$\begin{array}{l} \text{BRMS}{\text{B}}_{j}=\sqrt{{\left[QK^{-1}Q\right]}_{j,j}}, \end{array}$$

where $\text{BRMSE}\in {\mathbb{R}}^{N_{r}\times 1}$, $\text{SD}\in {\mathbb{R}}^{N_{r}\times 1}$, and $\text{BRMSB}\in {\mathbb{R}}^{N_{r}\times 1}$ are the Bayesian Root Mean Squared Error, Standard Deviation, and Bayesian Root Mean Squared Bias, respectively, and with ***K***^−1^ (and hence ***Q***, see Equation 17) defined using the prior hyper-parameters values estimated as described in Section 2.2.1.2. In this work, the estimation performance of different acquisition schemes is compared using the measures **SD** and **BRMSB** to separately quantify their estimation precision and accuracy, respectively, while the **BRMSE**, which incorporates both measures, is put forward as the decisive performance criterion.

#### 2.2.2. Monte Carlo validation

##### 2.2.2.1. Monte Carlo simulations

Monte Carlo experiments were performed to assess the generalizability of the BMSE-based metric results to brain images outside the training dataset (i.e., to ensure that the estimated prior did not overfit the brain image samples within the training dataset). To this end, *N*_*v*_ = 100 ground truth 2D HR images were extracted from a validation dataset generated as described in Section 2.2.1.1 and composed of brain images having different morphologies and contrasts than those within the training dataset.

First, noiseless MS images were simulated for the protocols HR, SRrot4 and SRsh4 using the MS-SRR forward model described by Equation (1). Next, the images were corrupted with additive white Gaussian noise, where the standard deviation of the noise for each image was chosen to correspond with its value assumed in the computation of the BMSE-based metrics. The conjugate gradient (CG) method (Hestenes and Stiefel, 1952) was used to solve the minimization problem described by Equation (8). The CG method optimization procedure is initialized at $0\in {\mathbb{R}}^{N_{r}\times 1}$ and stopped when the ratio between the 2-norm of the derivative of the cost function evaluated at the current iteration and at the initialization point is <10^−4^.

##### 2.2.2.2. Quantitative metrics

Monte Carlo estimates of the BMSE and its separate variance and squared bias components were computed, where the mean was calculated over *N*_*e*_ = 50 noise realizations for each of the *N*_*v*_ = 100 ground truth images included in the validation dataset, yielding:


(25) $${\hat{\text{BMSE}}}_{j}={\hat{\Sigma }}_{j,j}+\frac{1}{N_{v}}\sum_{n_{v}=1}^{N_{v}}{\left[\hat{\beta }\left(r^{\left(n_{v}\right)}\right){\hat{\beta }}^{T}\left(r^{\left(n_{v}\right)}\right)\right]}_{j,j},$$

(26) $$\begin{array}{l} {\hat{\Sigma }}_{j,j}=\frac{1}{N_{e}N_{v}}\overset{N_{v}}{\underset{n_{v}=1}{\sum }}\overset{N_{e}}{\underset{n_{e}=1}{\sum }}{\left({\hat{r}}_{j}^{\left(n_{e},n_{v}\right)}-\frac{1}{N_{e}}\overset{N_{e}}{\underset{n_{e}^{\prime }=1}{\sum }}{\hat{r}}_{j}^{\left(n_{e}^{\prime },n_{v}\right)}\right)}^{2}, \end{array}$$

(27) $$\begin{array}{l} \hat{\beta }\left(r^{\left(n_{v}\right)}\right)=\frac{1}{N_{e}}\overset{N_{e}}{\underset{n_{e}=1}{\sum }}{\hat{r}}^{\left(n_{e},n_{v}\right)}-r^{\left(n_{v}\right)}, \end{array}$$

with $r^{\left(n_{v}\right)}$ the *n*_*v*_-th ground truth image, and ${\hat{r}}^{\left(n_{e},n_{v}\right)}$ the *n*_*e*_-th MS-SRR estimate of $r^{\left(n_{v}\right)}$. Next, the Monte Carlo estimates of the BMSE-based metrics defined in Equations (22–24) were computed as:


(28) $${\hat{\text{BRMSE}}}_{j}=\sqrt{{\hat{\text{BMSE}}}_{j}},$$

(29) $$\begin{array}{l} {\hat{\text{SD}}}_{j}=\sqrt{{\hat{\Sigma }}_{j,j}}, \end{array}$$

(30) $${\hat{\text{BRMSB}}}_{j}=\sqrt{\frac{1}{N_{v}}\sum_{n_{v}=1}^{N_{v}}{\left[\hat{\beta }\left(r^{\left(n_{v}\right)}\right){\hat{\beta }}^{T}\left(r^{\left(n_{v}\right)}\right)\right]}_{j,j}}.$$

#### 2.2.3. Real data and retrospective simulation experiments

##### 2.2.3.1. Real data acquisition

Real data experiments were conducted for the acquisition protocols HR, SRrot_4_, and SRsh_4_ on a healthy volunteer after written informed consent in accordance with local ethics. The magnitude MS images were acquired in an interleaved fashion using a T2-weighted 2D FSE sequence on a Siemens Magnetom Prisma 3T system. TR/TE was set to 12,150/97 ms. The resolution was 1 × 1 × 1 mm^3^ for the HR protocol and 1 × 1 × 4 mm^3^ for the SRrot_4_ and SRsh_4_ protocols. The FOV was 256 × 256 × 192 mm^3^ for the SR protocols, while it was reduced to 256 × 256 × 128 mm^3^ for the HR protocol, since 128 was the maximum number of slices for acquisition allowed by the system. The acquisition time for a single MS image *t*_*acq*_/*N* and the total acquisition time *t*_*acq*_ for all the protocols are reported in Table 1. The HR protocol scan time is approximately equal to 2/3 of the SR protocols scan time, as only 2/3 of the field of view of the SR protocols is covered. GRAPPA with an acceleration factor of 2 was adopted in combination with the SMF technique, which is provided as “adaptive combine” on the employed MRI scanner. Two repetitions of the experiment (test-retest) were performed for each protocol.

###### 2.2.3.1.1. Image preprocessing

The preprocessing stage is composed of two steps: (i) intensity-normalization of the acquired MS images, (ii) correction of the acquired MS images for inter-image motion.

**Figure 2:**
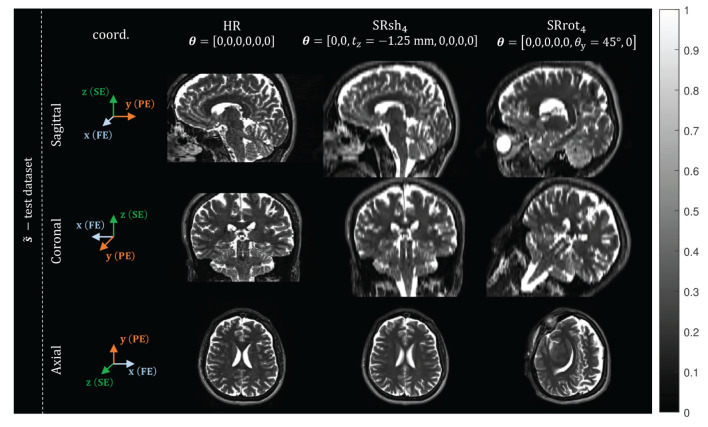
Examples of intensity-normalized acquired MS images: views from orthogonal planes of one of the MS images from the test dataset, for each protocol. The MR image coordinate system is reported in the first column, where FE, PE, and SE, represent the frequency-encoding (x-axis), phase-encoding (y-axis), and slice-encoding (z-axis) directions, respectively. For each MS image, the corresponding rigid transformation parameter vector θ is shown.

The purpose of the first step is to allow the use of the prior hyper-parameters values estimated from the synthetic training dataset on the acquired MR data. To this end, the voxel intensities of the MS-SRR image estimated from the acquired MS images have to be in the same range as the voxel intensities of the images within the synthetic training dataset. This can be achieved by normalizing the intensity of the acquired MS images, since the MS-SRR image preserves the intensity range of the images from which it is estimated. The intensity-normalization was performed using the white matter (WM) tissue as a reference. Four regions of interest (ROIs) were selected in the WM region of each of the acquired MS images for all the protocols. The mean WM intensity value was computed by averaging all the voxel intensities within the ROIs. A scaling factor was then applied so that the so-computed mean WM intensity matched the WM intensity value predicted by the model (Equation 21) used to generate the images within the training dataset. Examples of the intensity-normalized acquired MS images are represented in Figure 2.

**Figure 3:**
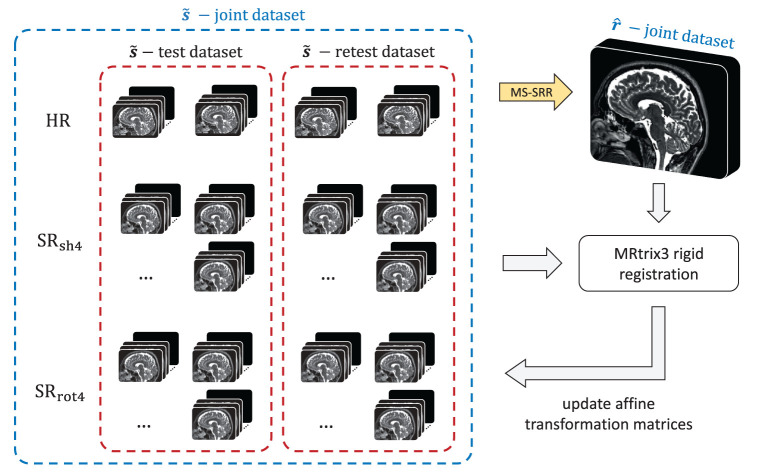
Workflow of the model-based iterative registration algorithm for joint motion correction of the MS images.

In the second step, all the acquired MS images from both the test and retest dataset were jointly corrected for rigid motion by using an iterative approach, presented in Figure 3. First, a MS-SRR image was estimated from all the MS images simultaneously by solving Equation (8) using the CG method, where the initialization point and stopping criterion were set as in Section 2.2.2. Then, a multi-scale 3D rigid registration between each MS image and the MS-SRR image was performed using the function “*mrregister”* from the MRtrix3 toolbox (RRID:SCR_006971; Tournier et al., 2019), and the affine transformation matrix of each MS image was updated. The procedure is iterated until the condition $||{\hat{\text{r}}}^{i}-{\hat{\text{r}}}^{i-1}|{|}_{2}<\text{tol}\left(1+||{\hat{\text{r}}}^{i}|{|}_{2}\right)$ was met, where ${\hat{\text{r}}}^{i}$ is the MS-SRR image estimated from all the acquired MS images in the *i*-th iteration, ||·||_2_ is the 2-norm operator, and the optimization tolerance tol was set to 0.01.

###### 2.2.3.1.2. Standard deviation of the noise and SNR

For each pair of pre-processed test-retest MS images, the standard deviation of the noise σ_*n*_ and the signal-to-noise ratio SNR_*n*_ were estimated for each protocol from the WM ROIs previously selected in the image pre-processing step as described in Section 2.2.3.1.1. The “difference method” described in Dietrich et al. (2007) was applied, yielding:


(31) $$\begin{array}{l} {\hat{\sigma }}_{n}=\frac{1}{\sqrt{2}}\sigma_{{\text{diff}}_{n}}, \end{array}$$

(32) $$\begin{array}{l} {\hat{\text{SNR}}}_{n}=\frac{\frac{1}{2}\mu_{{sum}_{n}}}{{\hat{\sigma }}_{n}}, \end{array}$$

with σ_*diff*_*n*__ the standard deviation of the *n*-th difference image in the ROIs and μ_*sum*_*n*__ the mean signal intensity value of the *n*-th sum image in the ROIs. Finally, the *N* estimates of the noise standard deviation and SNR were averaged to obtain single overall estimates $\hat{\sigma }$ and $\hat{\text{SNR}}$ for each protocol, which are reported in Table 1.

###### 2.2.3.1.3. 3D training dataset

The 3D training dataset, composed of 100 HR 3D T2-weighted brain images, was simulated from the 20 anatomical brain models available in the Brainweb database. Similarly to as described in Section 2.2.1.1 for the 2D case, T2-weighted brain volumes with different contrasts were generated from the anatomical models according to Equation (21) and subsequently down-sampled to a resolution of 1 × 1 × 1 mm^3^.

###### 2.2.3.1.4. 3D MRF prior hyperparameters

The prior hyperparameters for the real data experiments were learned from a training dataset composed of 100 noiseless HR 3D T2-weighted magnitude brain images of resolution 1 × 1 × 1 mm^3^. The generation of this 3D training dataset is described in Section 1 of the Supporting Information. The prior hyperparameters **α**, λ, and $\bar{r}$ were estimated from the image voxels within the 3D training dataset and their respective 3D neighborhoods using the approach described in Section 2.2.1.2. For the estimation of α and λ, a neighborhood size of 11×11×11 was selected, again using the same approach as in Section 2.2.1.2.

##### 2.2.3.2. Retrospective simulation experiments

The real data experiments described in Section 2.2.3 were replicated in retrospective simulations, using as ground truth the MS-SRR image estimated from all the pre-processed acquired MS images from all the protocols simultaneously. The acquisition process was simulated for the protocols HR, SRrot_4_ and SRsh_4_ using the MS-SRR forward model in Equation (1). The simulated images were corrupted with Rician noise. Additionally, patient motion was simulated as abrupt inter-image motion by modifying the rigid transformation parameter vector of the *n*-th MS image as follows (Ramos-Llordén et al., 2017):


(33) $$\begin{array}{l} \theta_{n}^{\prime }=\theta_{n}+d_{n}, \end{array}$$

where **θ**_*n*_ is the original rigid transformation parameter vector, $\theta_{n}^{\prime }$ is the motion-corrupted rigid transformation parameter vector, and $d_{n}\in {\mathbb{R}}^{6\times 1}$ is a vector-valued, zero mean, Gaussian random variable with covariance matrix $C=\sigma_{mot}^{2}I$, with σ_*mot*_ the standard deviation of each of the elements of ***d***_*n*_ and ***I*** ∈ ℝ^6×6^ the identity matrix. Different levels of motion were simulated by sampling σ_*mot*_ in the closed interval [0, 1]mm/°, with steps of 0.01mm/° between [0, 0.1], which will be referred to as the minor motion range, and with steps of 0.1 between (0.1, 1], which will be referred to as the major motion range, where $\sigma_{mot}=0\text{mm}{/}^{•}$ represents the motion-free scenario. The standard deviation of the noise and the prior hyper-parameters were set as in the real data experiments.

##### 2.2.3.3. Quantitative metrics

The MS-SRR images were estimated for each protocol and for each repetition of the experiment (*N*_*e*_ = 2, test-retest) in both real data and retrospective simulation experiments by solving Equation (8) using the CG method from the acquired and retrospectively simulated MS images, respectively, where the initialization point and stopping criterion were set as in Section 2.2.2. In the retrospective simulations, the MSE and its separate variance and squared bias components were estimated, where the mean was calculated over *N*_*e*_ = 2 repetitions of the experiment:


(34) $$\begin{array}{l} {\hat{MSE}}_{j}={\hat{\Sigma }}_{j,j}+{\left[\hat{\beta }\left(r\right){\hat{\beta }}^{T}\left(r\right)\right]}_{j,j}, \end{array}$$

(35) $$\begin{array}{l} {\hat{\Sigma }}_{j,j}=\frac{1}{N_{e}}\overset{N_{e}}{\underset{n_{e}=1}{\sum }}{\left({\hat{r}}^{\left(n_{e}\right)}-\frac{1}{N_{e}}\overset{N_{e}}{\underset{n_{e}^{\prime }=1}{\sum }}{\hat{r}}^{\left(n_{e}^{\prime }\right)}\right)}^{2}, \end{array}$$

(36) $$\begin{array}{l} \hat{\beta }\left(r\right)=\frac{1}{N_{e}}\overset{N_{e}}{\underset{n_{e}=1}{\sum }}{\hat{r}}^{\left(n_{e}\right)}-r, \end{array}$$

with ***r*** the ground truth image, and ${\hat{r}}^{\left(n_{e}\right)}$ the *n*_*e*_-th MS-SRR estimate of ***r***. MSE-based metrics were computed as:


(37) $${\hat{\text{RMSE}}}_{j}=\sqrt{{\hat{\text{MSE}}}_{j}},$$

(38) $$\begin{array}{l} {\hat{\text{SD}}}_{j}=\sqrt{{\hat{\Sigma }}_{j,j}}, \end{array}$$

(39) $${\hat{\text{RMSB}}}_{j}=\sqrt{{\left[\hat{\beta }\left(r\right){\hat{\beta }}^{T}\left(r\right)\right]}_{j,j}}.$$

where $\text{RMSE}\in {\mathbb{R}}^{N_{r}\times 1}$, and $\text{RMSB}\in {\mathbb{R}}^{N_{r}\times 1}$ are the Root Mean Squared Error, and Root Mean Squared Bias, respectively. **RMSE** is a combined measure of accuracy and precision, while **RMSB** quantifies the estimation accuracy only. The median **RMSE**, **SD**, and **RMSB** values were computed from the voxel values of each respective map within a brain mask. The brain mask was calculated using the Brain Extraction Tool (BET) of the FSL toolbox (RRID:SCR_002823; Woolrich et al., 2009) from the MS-SRR image estimated from all the pre-processed MS images acquired adopting the HR protocol simultaneously. In the real data experiments, only the estimation precision **SD** could be assessed due to the absence of a ground truth.

## 3. Results

### 3.1. MS-SRR protocols comparison in terms of BMSE

**Figure 4:**
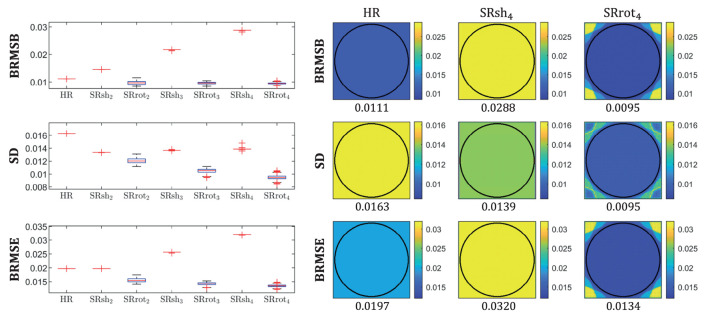
Boxplots of BRMSE, SD, and BRMSB computed for each protocol inside an ROI **(left)** and the respective parametric maps **(right)** for the protocols HR, SRrot_4_, and SRsh_4_. For each box, the central mark indicates the median, and the bottom and top edges of the box indicate the 25th and 75th percentiles, respectively, and the outliers are plotted individually using the “+” marker symbol (MATLAB, 2020). Each box has a sample size of 35,629 voxels. For each map, the median value computed inside an ROI is reported. The ROI was defined as the part of the field of view common to all the acquired images of all the acquisition protocols, which corresponds with the area inside the circle plotted in the maps.

The BRMSB, SD, and BRMSE maps computed using the closed-form expressions (Equations 22–24) and their distributions inside an ROI are reported in Figure 4, where the ROI was defined as the part of the FOV that is common to all the MS images of all the MS-SRR acquisition protocols included in the comparison. The median values were computed for each distribution and reported below the respective maps and in Table 2.

**Table 2:** Quantitative comparison results for the HR, SRsh_4_, and SRrot_4_ protocols.

	**HR**	**SRsh_4_**	**SRrot_4_**
**Analytical calculations (2D)**			
BRMSB	0.0111	0.0288	0.0095
SD	0.0163	0.0139	0.0095
BRMSE	0.0197	0.0320	0.0134
**Monte Carlo simulations (2D)**			
$\hat{\text{BRMSB}}$	0.0113	0.0278	0.0097
$\hat{\text{SD}}$	0.0160	0.0136	0.0093
$\hat{\text{BRMSE}}$	0.0198	0.0310	0.0135
**Real data experiment (3D)**			
$\hat{\text{SD}}$	0.0121	0.0148	0.0084
**Retrospective simulations—no motion (3D)**			
$\hat{\text{RMSB}}$	0.0167	0.0262	0.0138
$\hat{\text{SD}}$	0.0062	0.0047	0.0043
$\hat{\text{RMSE}}$	0.0197	0.0272	0.0156
**Retrospective simulations—minor motion (3D)**			
$\hat{\text{RMSB}}$	0.0184	0.0357	0.0158
$\hat{\text{SD}}$	0.0100	0.0145	0.0076
$\hat{\text{RMSE}}$	0.0245	0.0462	0.0203
**Retrospective simulations—major motion (3D)**			
$\hat{\text{RMSB}}$	0.0584	0.1725	0.0622
$\hat{\text{SD}}$	0.0377	0.1396	0.0371
$\hat{\text{RMSE}}$	0.0855	0.2854	0.0850

The first section of the table reports the median values of the BMSE-based metrics, computed adopting the respective closed forms derived in Section 2.1.3. The second section reports the median Monte Carlo estimates values of the BMSE-based metrics. The third section reports the median SD values estimated from real data experiments. The last section reports the median RMSB, SD, and RMSE values estimated from retrospective simulations experiments for different levels of inter-image motion (no motion: σ_*mot*_ = 0, minor motion: σ_*mot*_ = 0.1, and major motion: σ_*mot*_ = 1). The lowest value for each metric, which indicates the protocol with the best estimation performance in terms of that metric, is reported in bold.

BRMSB quantifies the estimation accuracy of the protocols, where a lower BRMSB value corresponds with a higher estimation accuracy. It follows from Figure 4 that the SRrot protocols provide a slightly lower BRMSB value than the reference protocol, whereas the BRMSB value provided by the SRsh protocols is substantially larger than that of the HR reference protocol. For the SRsh protocols, BRMSB increases for increasing values of AF up to a factor of 2.7 difference between SRsh_4_ and HR, while it slightly decreases with AF for the SRrot protocols. SD quantifies the estimation precision of the protocols, where a lower SD value corresponds with a higher precision. Both the SRrot and SRsh protocols provide a lower SD than the reference protocol HR, with the SRrot protocols outperforming the SRsh protocols. For the SRrot protocols, the SD decreases for increasing values of AF up to a factor of 1.7 difference between SRrot_4_ and HR, while it slightly increases with AF for the SRsh protocols. On balance, the accuracy and precision components together result in higher BRMSE values for the SRsh protocols and substantially lower BRMSE values for the SRrot protocols, compared to the reference protocol HR. For the SRsh protocols, BRMSE increases for increasing values of AF up to a factor of 1.7 difference between SRsh_4_ and HR. For the SRrot protocols, on the other hand, BRMSE decreases with AF up to a factor of 1.5 difference between SRrot_4_ and HR. In conclusion, the SRrot protocols outperform the competing protocols in terms of BRMSE, SD, and BRMSB.

### 3.2. Monte Carlo validation

**Figure 5:**
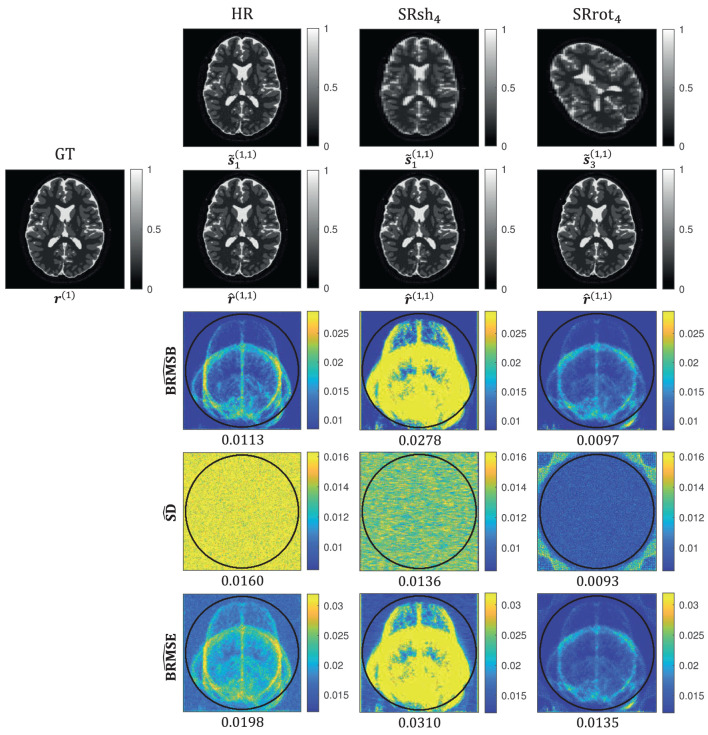
Monte Carlo simulation results for the protocols HR, SRrot_4_, and SRsh_4_. In the first two rows, $r^{\left(n_{v}\right)}$, ${\tilde{s}}_{n}^{\left(n_{e},n_{v}\right)}$, and ${\hat{r}}^{\left(n_{e},n_{v}\right)}$ represent the *n*_*v*_-th image from the validation dataset, the *n*-th noise-corrupted MS image simulated using the *n*_*v*_-th image from the validation dataset as ground truth in the *n*_*e*_-th repetition of the experiment, and the MS-SRR image estimated from ${\tilde{s}}^{\left(n_{e},n_{v}\right)}$, respectively. In the last three rows, the Monte Carlo estimates $\hat{\text{BRMSB}}$, $\hat{\text{SD}}$, and $\hat{\text{BRMSE}}$ are shown. For each map, the median value computed inside an ROI is reported. The ROI was defined as the part of the field of view common to all the acquired images of all the acquisition protocols, which corresponds with the area inside the circle plotted in the maps.

Monte Carlo estimates of the BMSE-based metrics computed using the expressions in Equations (25–30) for the protocols HR, SRrot_4_, and SRsh_4_ are presented in Figure 5. The median values computed inside the ROI are reported below the maps as well as in Table 2, where the ROI corresponded with the one visualized in Figure 4. These median values are in agreement with the median values computed from the BRMSB, SD, and BRMSE maps that were reported in Section 3.1.

### 3.3. Real data and retrospective experiments

#### 3.3.1. Real data experiments

**Figure 6:**
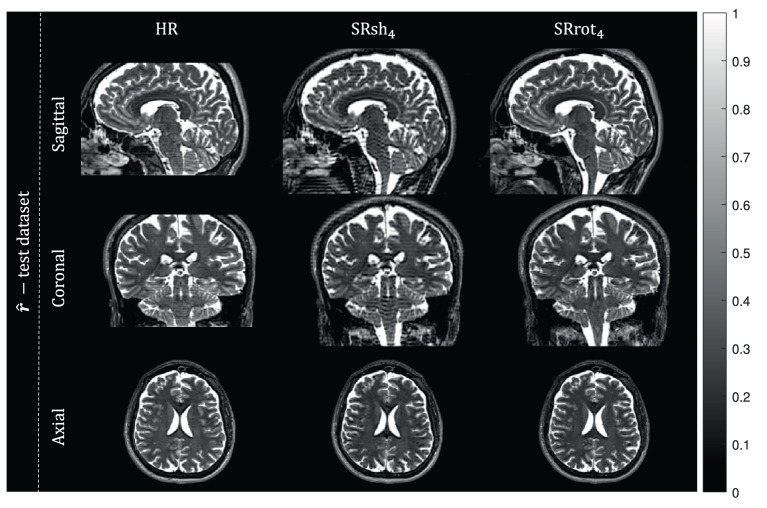
Real data results: MS-SRR images. The views from orthogonal planes of the MS-SRR images estimated from the test dataset for each protocol are shown.

**Figure 7:**
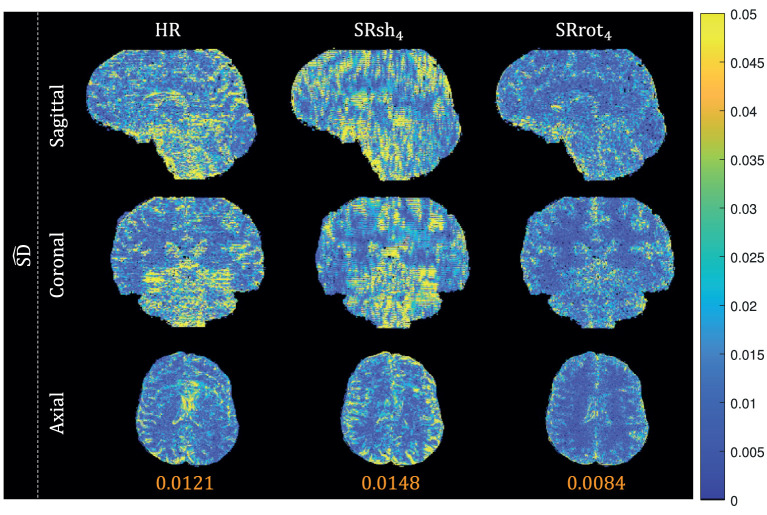
Real data results: SD maps. The views from orthogonal planes of the SD map for each protocol are shown, and the median value computed inside a brain mask for each SD map is reported.

Orthogonal views of the MS-SRR image estimated for each protocol from the acquired MS images of the first repetition of the experiment are reported in Figure 6. Reconstruction artifacts in the slice-encoding direction are noticeable in the MS-SRR image estimated from the shifted MS images. The SD maps calculated for each protocol from the MS-SRR images of the two repetitions of the experiment are reported in Figure 7 and Table 2. The median SD was computed from the voxels inside a brain mask for each protocol, computed as described in Section 2.2.3.3. It follows from Figure 7 that SRrot_4_ provides the highest estimation precision among the acquisition protocols included in the comparison, followed by the HR protocol and the SRsh_4_ protocol, in descending order.

#### 3.3.2. Retrospective simulation experiments

**Figure 8:**
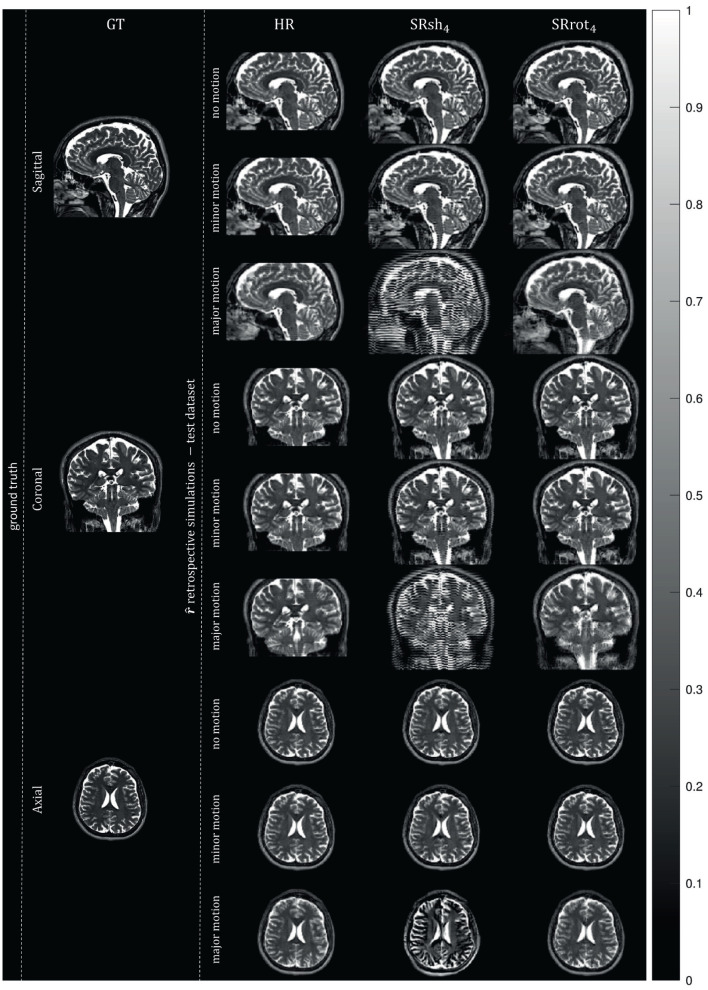
Retrospective simulation results: MS-SRR images. The views from orthogonal planes of the ground truth image, which corresponds to the MS-SRR image estimated from all the pre-processed acquired MS images from all the protocols simultaneously, and the MS-SRR images estimated from the test dataset for each protocol in the absence of motion (σ_*mot*_ = 0 mm/°) and in the presence of motion levels equal to the upper bounds of the minor (σ_*mot*_ = 0.1 mm/°) and major (σ_*mot*_ = 1 mm/°) motion ranges are shown.

**Figure 9:**
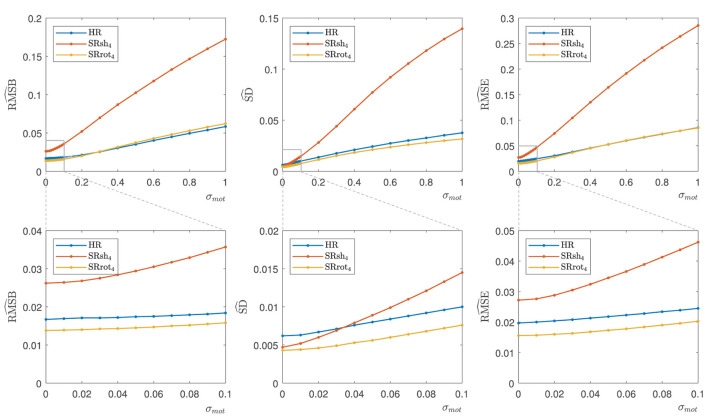
Retrospective simulation results: $\hat{\text{RMSB}}$, $\hat{\text{SD}}$, and $\hat{\text{RMSE}}$ median values. The median values were calculated for each parameter from the respective parameter map within a brain mask in the absence of motion (σ_*mot*_ = 0) and in the presence of minor (0 mm${/}^{•}\leq \sigma_{mot}\leq 0.1$ mm/°) and major (0.1 mm${/}^{•}\leq \sigma_{mot}\leq 1$ mm/°) levels of inter-image motion. The bottom row shows a zoomed plot corresponding to the indicated area.

Orthogonal views of the MS-SRR image estimated for each protocol from the retrospectively simulated MS images are reported in Figure 8 in the absence of inter-image motion (σ_*mot*_ = 0 mm/°) and in the presence of minor (σ_*mot*_ = 0.1 mm/°) and major (σ_*mot*_ = 1 mm/°) levels of inter-image motion. In the absence of inter-image motion, the MS-SRR images from the HR, SRsh_4_, and SRrot_4_ protocols are visually comparable. In the presence of a minor level of inter-image motion, inter-slice intensity artifacts can be found in the SRsh_4_ MS-SRR image, whereas the HR and SRrot_4_ MS-SRR images are not visibly impacted. In the presence of a major level of inter-image motion, the motion artifacts for the SRsh_4_ MS-SRR image become so severe that they impede the delineation of the underlying brain structures. Inter-slice intensity artifacts and blurring artifacts caused by motion also start to appear in the HR and SRrot_4_ MS-SRR images, respectively, but they are not as severe as the artifacts present in the SRsh_4_ MS-SRR image. Figure 9 shows the median values within a brain mask of the RMSB, SD, and RMSE maps that were computed for the protocols HR, SRsh_4_, and SRrot_4_ in the absence of motion and for the minor and major motion ranges. In the absence of inter-image motion, the relative performance of the different protocols with respect to each other in terms of RMSB, SD and RMSE is as predicted by the BMSE-based analysis that was reported in Section 3.1. However, in the minor motion range, the estimation precision for SRsh_4_ significantly decreases (i.e., SD increases), leading SRsh_4_ to be outperformed by HR in terms of estimation precision for σ_*mot*_ ≥ 0.04 mm/°, as observed in the real data experiments. The SRrot_4_ protocol showed instead consistent performance in the minor motion range, outperforming SRsh_4_ and HR in terms of the RMSB, SD, and RMSE. Finally, in the major motion range, SRrot_4_ showed a lower estimation accuracy (i.e., higher RMSB) than the HR protocol for σ_*mot*_ ≥ 0.3 mm/°, whereas SRrot_4_ kept showing a higher estimation precision (i.e., lower SD) than HR, leading to overall comparable results for SRrot_4_ and HR in terms of RMSE.

## 4. Discussion

In this work, a Bayesian framework was proposed to compare two commonly adopted MS-SRR acquisition strategies, i.e., the rotated scheme and the shifted scheme, in terms of their estimation performance. This comparison is novel since it allows to include the effect of prior knowledge-based regularization, thereby extending previous work of Shilling et al. (2008, 2009), which focused on non-regularized MS-SRR.

It was shown that the MAP estimator provides higher accuracy and precision, and a lower MSE when applied to data acquired with the rotated scheme than when applied to data acquired with the shifted scheme or the reference scheme that consists of a conventional HR MS acquisition, where the acquisition time for all schemes was equal. This finding was based on analytical calculations of BMSE-based metrics (cfr. Figure 4 and Table 2), which were confirmed by Monte Carlo simulations (cfr. Figure 5), and was supported by real-data experiments (cfr. Figures 6, 7 and Table 2). Furthermore, both real data experiments and retrospective simulation results highlighted a higher resilience to motion for the rotated and reference schemes compared to the shifted scheme (cfr. Figures 8, 9).

A possible explanation for the superior performance of the rotated scheme compared to the shifted scheme is that it allows a more effective sampling of the k-space. Indeed, since a rotation in image space results in a rotation in the frequency domain, the narrow slice selection band is oriented in a different direction of the frequency spectrum of the MS-SRR image to be reconstructed for each MS image. As a result, the dataset acquired with the rotated scheme contains high spatial frequencies in all dimensions. Conversely, when the shifted scheme is adopted, each MS image samples the same part of the k-space, and the MS-SRR estimation relies only on recovering the aliased frequency content by increasing the sampling density in the slice-encoding direction. Additionally, the superior performance of the rotated scheme compared to the reference scheme confirms the conclusions of Plenge et al. (2012) that, by adopting the rotated MS-SRR scheme, it is possible to achieve an improved trade-off between acquisition time, spatial resolution, and estimation precision compared to MS protocols based on HR acquisitions.

The quantitative agreement among the median values of the BMSE-based metrics and their respective Monte Carlo estimates observed in Table 2 suggests that the statistics of the images of the validation dataset were well described by the prior distribution whose parameters were estimated from the images of the training dataset, even though the images of the validation dataset may have a different contrast and morphology than the images of the training dataset. This shows that the validity of the BMSE-based analysis in this work extends to a more general class of brain images than those used for training. It should be noted that the visual differences between the BRMSB and BRMSE maps in Figure 4 and their respective Monte Carlo estimates $\hat{\text{BRMSB}}$ and $\hat{\text{BRMSE}}$ in Figure 5 were expected since the brain images within the validation dataset represent only a specific subset of the images described by the prior. Indeed, the $\hat{\text{BRMSB}}$ and $\hat{\text{BRMSE}}$ maps visually resemble the superposition of brain slices from the sagittal, axial, and transversal planes that compose the validation dataset.

Contrary to what was predicted by the BMSE-based metrics, a lower estimation precision for the shifted scheme was found compared to the reference scheme in the real data experiments results. This discrepancy can be attributed to the high sensitivity to motion of the shifted scheme compared to the other acquisition schemes. Indeed, we have observed from the retrospective simulations how even a small amount of motion (e.g., residual uncorrected motion among the MS images after the registration step) can lead to a significant decrease in precision (i.e., increase in SD) for the shifted scheme, which in turn leads the reference scheme to outperform the shifted scheme in terms of estimation precision (Figure 9, σ_*mot*_ ≥ 0.04). Additionally, the retrospective simulations show the shifted scheme MS-SRR images to be more severely impacted by motion artifacts than the rotated and reference schemes MS-SRR images, especially in the presence of major levels of motion (Figure 8). These motion artifacts can be mitigated by increasing the amount of regularization, but at the cost of reduced sharpness (i.e., lower resolution) of the MS-SRR image.

The presented results provide new insights for the optimization of the acquisition design of MS-SRR experiments. For example, in recent works (Hutter et al., 2017; Bastiani et al., 2019; Christiaens et al., 2021), super-resolution diffusion MRI frameworks based on the MS-SRR shifted scheme have been proposed to address neonatal MRI-specific issues, which include the high occurrence of image artifacts induced by the presence of severe motion. Our results suggest that using the rotated scheme instead of the shifted scheme in these frameworks would increase the resilience to such motion artifacts and lead to more accurate and precise MS-SRR estimates.

In this study, the image registration was performed (or assumed to be performed, in the case of the BMSE analytical calculations and MC simulations) as a preprocessing step prior to the actual MS-SRR step. Alternatively, MS-SRR methods have been proposed in the literature that allow the joint estimation of motion parameters along with the MS-SRR image in a unified approach (Shen et al., 2007; Fogtmann et al., 2012; Rezayi and Seyedin, 2017; Beirinckx et al., 2020, 2022). The impact of combining MS-SRR with integrated motion estimation on the accuracy and precision of the MS-SRR image needs further research but is not expected to change the main conclusions of this work.

Furthermore, the use of the proposed BMSE metrics was limited to the comparison of the shifted and rotated MS-SRR acquisition schemes. However, it may well be that there exists an optimal set of acquisition parameters for an MS-SRR experiment beyond these conventional schemes. In future work, we intend to further exploit the proposed Bayesian framework to find the optimal acquisition settings that minimize the estimation error of the MS-SRR image in a statistically well-defined way (Fedorov, 1972, 2010; Chaloner and Verdinelli, 1995). Finally, we intend to extend this approach to MS quantitative SRR (MS-qSRR), which differs from conventional MS-SRR in that HR 3D isotropic parametric maps are estimated instead of a weighted image (Van Steenkiste et al., 2015, 2016; Bano et al., 2019; Beirinckx et al., 2022).

This paper addressed the question: “What is the optimal acquisition strategy for regularized MS-SRR MRI in terms of estimation accuracy and precision? To shift or to rotate?.” To answer this question, a Bayesian framework was developed to compare two commonly applied MS-SRR acquisition schemes, denoted as the shifted and rotated scheme, respectively. In the shifted scheme, the MS images are shifted by different sub-pixel distances in the through-plane direction, whereas in the rotated scheme, the slice orientations are rotated around the phase-encoding axis by different angles. The rotated scheme was shown to outperform the shifted scheme in terms of the accuracy, precision, and mean squared error of the Bayesian MAP estimator. Furthermore, it was shown to be more robust to motion artifacts. Finally, unlike the shifted scheme, the rotated scheme showed an improved trade-off between scan time, spatial resolution, and estimation precision compared to a conventional MS protocol based on direct HR acquisition. The proposed Bayesian framework could be further exploited in optimal experimental design studies to find the acquisition settings of an MS-SRR experiment that minimize the estimation error in a statistically well-defined way.

## Data availability statement

The raw data supporting the conclusions of this article will be made available by the authors, without undue reservation.

## Ethics statement

Ethical review and approval was not required for the study on human participants in accordance with the local legislation and institutional requirements. The patients/participants provided their written informed consent to participate in this study.

## Author contributions

MN, BJ, DP, JS, and AdD conceived and designed the study. MN and CS performed the magnetic resonance imaging scanning. MN, BJ, QB, and DP wrote the MATLAB code. MN conducted the data analysis and wrote the first draft of the manuscript. All authors contributed to the writing, critical review, and approval of the final manuscript.

## Funding

This work is part of the project B-Q MINDED which has received funding from the European Union's Horizon 2020 research and innovation programme under the Marie Skłodowska-Curie grant agreement No. 764513. The authors gratefully acknowledge support from the Research Foundation Flanders (FWO) through project funding G084217N and 12M3119N.

## Conflict of interest

Author CS was employed by the company Siemens Healthcare NV/SA. The remaining authors declare that the research was conducted in the absence of any commercial or financial relationships that could be construed as a potential conflict of interest.

## Publisher's note

All claims expressed in this article are solely those of the authors and do not necessarily represent those of their affiliated organizations, or those of the publisher, the editors and the reviewers. Any product that may be evaluated in this article, or claim that may be made by its manufacturer, is not guaranteed or endorsed by the publisher.
